# Editorship of the Journal

**Published:** 1846-03

**Authors:** 


*Editorship of the Journal.*—It has hitherto been deemed policy to have the editors of the American Journal of Dental Science located in different parts of the country, and there are some good reasons why this should be so; although there are some objections to it. The time and attention which each of the editors has necessarily expended upon this publication, have hitherto been almost purely a donation to the society, and the interests of the profession, and it cannot, therefore, be expected that all other business could be laid aside to prepare articles for its pages. This not only accounts for the delay which has sometimes occurred in issuing it promptly, but connected with the fact that but one of the editors resides where the Journal is published, it serves also to account for errors which could be avoided, did each read his own proof.

This remark is particularly true as applied to the two articles which we furnished for the last Journal—the one upon the use of arsenic, and the other pertaining to the resolutions of the American Society, relative to the use of amalgams, &c.

We did not intend, until this number was nearly due, to furnish any thing for it. At this time we read a letter from Dr. Harris, advising us that his other engagements were such that he should expect us to prepare and send on immediately, one or more articles. This, at a very late date, we accomplished, but not in time to have the Journal issued when due, much less to enable us to get a proof of these articles. They were, therefore, both published as they best could be, without the manuscript even being copied, or any proof (by us at least) being read. We do not intend by these remarks, to cast any reflections upon the printer, as they were more or less cut up with interlineations and *obliterations*, but simply to account for, if not excuse, several errors which appeared in them.

A very striking example will be found on page 119, 13th line from bottom, where the word *inflamed* is used for *un*inflamed.

On page 169, 5th line from top, the word *faith* should be *part.* On the same page, 10th line from top, the word *however* should be inserted between the words *cases* and *than.* 21st line from top, the word *them* should be *their.* In the 11th line from bottom, same page, the word *objections* should be *objectors.* On page 171, the proper name written *Procostes,* is referred to the bottom of the page, and its orthography changed. Although this change was made in the manuscript, it was by no means intended for publication in this way. In looking over the manuscript hastily, this word struck our attention, and it was thus referred to the bottom of the page, with a query, to call Dr. Harris' attention to it, on whom we have generally relied to read the proof, he being on the spot, and having the immediate supervision of the Journal.

We can only say, in reference to this change, that we regret its occurrence in the Journal, and trust that our explanation will be satisfactory to the gentleman in whose writing it occurred. Page 178, 17th line from bottom there is a typographical error in the word intended for Mallan. Had any of the above inaccuracies had reference to any but ourself, we should not have noticed them specially. W.

